# Diffracting molecular matter-waves at deep-ultraviolet standing-light waves†

**DOI:** 10.1039/d4cp03059a

**Published:** 2024-10-22

**Authors:** Ksenija Simonović, Richard Ferstl, Alfredo Di Silvestro, Marcel Mayor, Lukas Martinetz, Klaus Hornberger, Benjamin A. Stickler, Christian Brand, Markus Arndt

**Affiliations:** a University of Vienna, Faculty of Physics, VDS VCQ, Boltzmanngasse 5 1090 Vienna Austria ksenija.simonovic@univie.ac.at markus.arndt@univie.ac.at; b Department of Chemistry, University of Basel St. Johannsring 19 4056 Basel Switzerland; c University of Duisburg-Essen Lotharstraße 1 47048 Duisburg Germany; d Ulm University, Institute for Complex Quantum Systems and Center for Integrated Quantum Science and Technology Albert-Einstein-Allee 11 89069 Ulm Germany; e German Aerospace Center (DLR), Institute of Quantum Technologies Wilhelm-Runge-Straße 10 89081 Ulm Germany

## Abstract

Matter-wave interferometry with molecules is intriguing both because it demonstrates a fundamental quantum phenomenon and because it opens avenues to quantum-enhanced measurements in physical chemistry. One great challenge in such experiments is to establish matter-wave beam splitting mechanisms that are efficient and applicable to a wide range of particles. In the past, continuous standing light waves in the visible spectral range were used predominantly as phase gratings, while pulsed vacuum ultraviolet light found applications in photoionization gratings. Here, we explore the regime of continuous, intense deep-ultraviolet (> 1 MW cm^−2^, 266 nm) light masks, where a rich variety of photo-physical and photo-chemical phenomena and relaxation pathways must be considered. The improved understanding of the mechanisms in this interaction opens new potential pathways to protein interferometry and to matter-wave enhanced sensing of molecular properties.

## Abstract 2

We demonstrate an optical 266 nm beamsplitter for molecular matter-waves, observing effects of optical molecular properties in the gas phase such as absorption cross section and polarisability.

## Graphical abstract

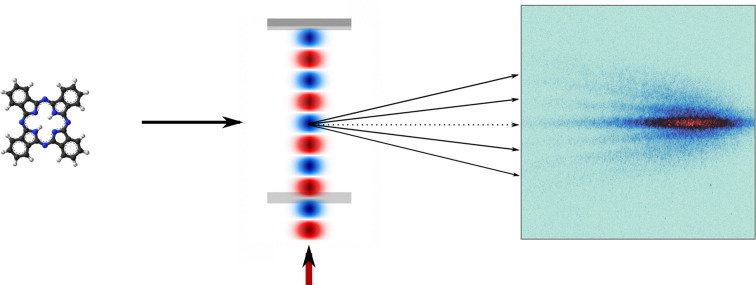

## 1 Introduction

Shortly after Louis de Broglie's prediction that one needs to ‘associate a periodical phenomenon with any isolated portion of matter or energy’ and that we should see this ‘in phase with a wave’,^1^ matter waves were experimentally confirmed for electrons,^2,3^ neutral He atoms and H_2_ molecules,^4^ as well as for neutrons.^5^ De Broglie's revolutionary proposal about the wave behaviour of matter^1^ started the theoretical formulation of modern quantum physics^6^ and quantum chemistry, where this idea is at the heart of molecular bond and orbital theory.^7,8^ While in chemistry electron quantum waves are usually confined inside an atom or molecule, a whole research field has evolved around the question of how to describe the center-of-mass motion of single and composite systems, from electrons^9^ over neutrons^10^ and atoms^11,12^ to complex molecules^13^ or even antimatter.^14^

Here, we are focusing on new tools for quantum coherent manipulation of the center-of-mass motion of large molecules, inspired by advances in atom interferometry and progress in the diffraction of cold dimers,^15^ small noble gas clusters,^16,17^ and large molecules.^18^ Numerous molecule interferometers have been built throughout the last two decades to explore molecular transition strengths,^19,20^ to study the quantum wave nature of fullerenes,^21^ vitamins,^22^ polypeptides,^23^ clusters of organic molecules^24^ or tailor-made compounds with masses even beyond 25 kDa.^25^ A variety of recent experiments in physical chemistry have focused on the analysis of molecules and clusters in classical and quantum beam deflectometry.^26–31^ These studies find a valuable complement in matter-wave interferometry which also allows measuring the electric,^32^ magnetic,^33^ optical^34^ or structural properties^32,35,36^ of complex molecules *via* deflection of fine-grained quantum interference fringes.

Extending matter-wave interferometry to an even larger set of molecules requires new methods for molecular beam generation, beam splitters, and efficient single-molecule detectors. Here, we focus on how to realize deep ultraviolet beam splitters and how they interact with the rich set of internal molecular properties. Inspired by early achievements in atom optics,^37,38^ nanomechanical masks have already been successfully used to manipulate molecular beams.^18,39–41^ Although these nanostructures are very well suited for many atoms and molecules with low electric polarizability and dipole moments,^42,43^ optical gratings cannot be clogged or destroyed. They are perfectly periodic, adjustable *in situ* and in real time and they may also exploit internal states that nanomasks would not be sensitive to.

Inspired by previous experiments in atom optics^44–46^ and electron optics,^47^ optical phase gratings were realized for molecular beams of fullerenes^48^ and even antibiotics^49^ and pulsed vacuum-ultraviolet photoionization gratings as matter-wave beam splitters for organic clusters^24^ and polypeptides.^23^ Here, we study the regime of continuous, high-intensity deep-ultraviolet (DUV) light masks. The wavelength of *λ*_L_ = 266 nm is close to a (usually very broad) electronic transition in many aromatic molecules and high-power laser light can be generated with high coherence and in a good beam profile by second harmonic generation of a diodepumped solid state laser. For thermal beams of molecules with an absorption cross section around *σ*_abs_ ≃ 10^−16^ cm^2^ and velocities in the range of 100–300 m s^−1^, laser intensities around 1 MW cm^−2^ are required to ensure that selected chromophores absorb one or a few photons during their transit through the laser beam. Here, we demonstrate the realization of such optical gratings and discuss how the internal state evolution after the absorption process influences the evolution of the quantum wave that is associated with the molecular center-of-mass motion.

**Fig. 1:**
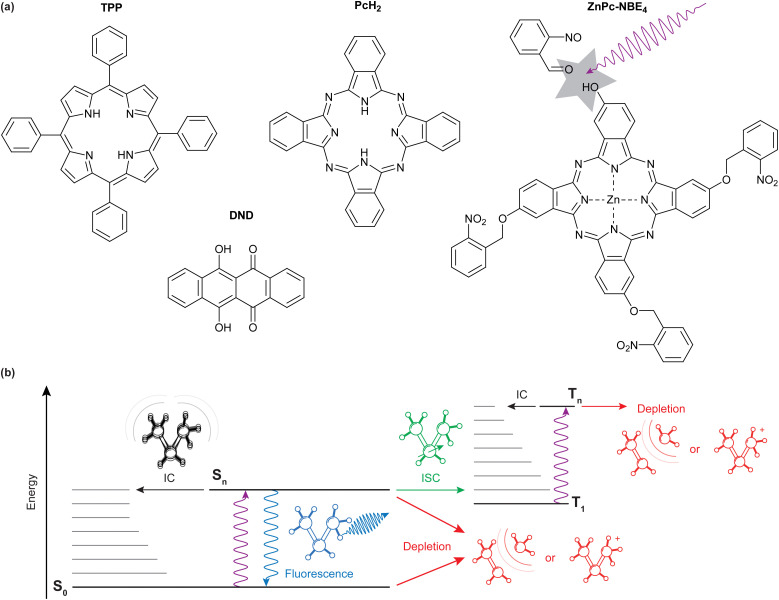
(a): Molecules explored in this experiment, from left to right: *meso*-Tetraphenylporphyrin (TPP), 6,11-dihydroxy-5,12-naphthacenedione (DND), metal-free phthalocyanine (PcH_2_), a zinc-coordinated derivative of phthalocyanine (ZnPc–NBE_4_). (b): Possible internal relaxation pathways after deep-ultraviolet photoexcitation. After electronic excitation, the emission of a fluorescence photon adds a randomly oriented photon recoil to the molecule, blurring the respective diffraction peaks. This is not the case for nonradiative processes, such as internal conversion and intersystem crossing. Fragmentation or ionization may occur from any excited state or a hot ground-state molecule, effectively removing it from the beam.

We specifically compare the four molecules shown in Fig. 1(a): *meso*-tetraphenylporphyrin (TPP, *m* = 614.7u), 6,11-dihydroxy-5,12-naphthacenedione (DND, and *m* = 290.3u), phthalocyanine (PcH_2_, *m* = 514.5u) and a zinc-coordinated phthalocyanine where each isoindole unit is bound to an *ortho*-nitro benzylic ether (NBE) group as a photocleavable tag (ZnPc–NBE_4_, *m* = 1182.4u). TPP, DND, and PcH_2_ were obtained commercially (Sigma Aldrich/Merck) and used without further purification while ZnPc–NBE_4_ was synthesized by us based on a phthalocyanine core (see ESI†). We use these systems to explore the role of different molecular energy relaxation pathways, some of which are indicated in the level scheme of Fig. 1(b). They include internal conversion (IC), intersystem crossing (ISC), fluorescence, and the bond dissociation of a photocleavable tag. We discuss how these internal effects influence the de Broglie wave, *i.e.* the quantum evolution of the molecular center-of-mass motion, and how to observe it in experiments.

## 2 Experimental setup

**Fig. 2:**
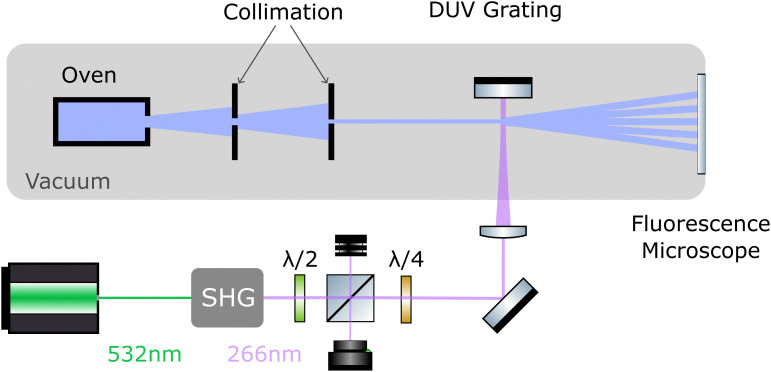
Experimental setup: A thermal molecular beam is collimated to a divergence of 5 μrad to approximate a plane-parallel matter wave. The molecules are diffracted at a deep ultraviolet grating which is generated as a standing light wave of a high-power continuous frequency doubled laser. The diffracted molecules generate a mass density pattern on the window of that vacuum chamber, which is imaged by fluorescence microscopy. During diffraction, the matter-wave beam splitter imparts a transverse momentum of Δ*p* = ±*nħk*_L_, with the integer *n* depending on the details of the process.

The layout of the experiment is shown in Fig. 2. All molecules are sublimated in a thermal source, and the resulting beam is collimated to an angle below 5 μrad. Molecules of different velocity are spatially dispersed by their free-flight parabolas with a 20 μm high delimiter placed immediately behind the grating (not shown). This slit additionally ensures that all detected molecules have interacted with the light grating. The molecules propagate another 0.7 m until they reach a thin quartz slide at the end of the vacuum chamber, where they are imaged using laser-induced fluorescence microscopy.^50^

### 2.1 UV diffraction grating

To realize the standing wave laser grating, 5 W of laser radiation at *λ*_L_ = 532 nm is frequency doubled in an external resonator (Sirah Wavetrain 2) to *λ*_L_ = 266 nm with an output power of about 1.2 W. The DUV light is focused onto a dielectric mirror in high vacuum (1 × 10^−7^ mbar), with its surface aligned parallel to the molecular beam. To protect the laser from back-reflected light, and to control the grating power, we employ an optical isolator, consisting of a *λ*/2 plate in front of a polarizing beam splitter and a *λ*/4 plate behind it. The light in the optical grating is therefore circularly polarized. We track the power of the retroreflected DUV beam and find that it is stable to within 10% during a measurement run. However, irradiating the mirror with light intensities beyond 1 MW cm^−2^ at 1 × 10^−7^ mbar leads to a slow degradation of the mirror surface. To compensate for this, we shift the mirror parallel to the molecular beam in between measurements to expose a fresh spot to the laser. Given a grating period of *λ*_L_/2 = 133 nm and a laser waist of 12–15 μm,^51^ the molecular beam divergence and its inclination to the mirror surface have to be smaller than 1 mrad, to ensure that all molecules see a well-defined optical grating.

### 2.2 Imaging of the diffraction patterns

The interference patterns land softly on a quartz slide at the end of the vacuum chamber where they are illuminated by a homogeneous diffuse laser beam. TPP is excited at 421 nm, DND at 266 nm, and PcH_2_ as well as ZnPc–NBE_4_ by 661 nm laser light. The fluorescence band pass filters are 630 nm to 670 nm for TPP, 506 nm to 594 nm for DND, 698.5 nm to 723.5 nm for PcH_2_ and 672 nm to 712 nm for ZnPc–NBE_4_. The fluorescence signal was integrated for five minutes. The imaging system consists of a 20× Zeiss plan neofluoar objective, a tube lens of (*f* = 164 mm) and an Andor iXON 3 EMCCD camera, cooled to −75 °C. We do not observe significant fluorescence bleaching except for DND. We corrected the raw images by subtracting both the signal without the detection laser as well as illuminated images taken before the molecule deposition from the raw data. This reduces the contribution of stray light and dust particles. Some obvious contamination spots were manually removed and the intensity spikes were flattened by removing the lowest and highest 10^−5^-quantile of the data set. The effect of inhomogeneities in the ambient light is reduced by subtracting a linear fit to the image, gained by interpolating between the regions outside the diffraction pattern. Additionally, we corrected for a rotation of the camera with respect to gravity.

### 2.3 Simulation of the diffraction patterns

While many aspects of matter-wave diffraction can be surprisingly well described using undergraduate-level mathematics,^52^ accounting for all experimental details and molecular processes requires a full quantum description. Our model accounts for the interaction between the molecules and the optical grating, the role of finite coherence and decoherence, the source collimation and velocity distribution, and many internal relaxation pathways. The complete theory is based on propagating Wigner functions, as described in a separate paper.^53^ Details of the simulation parameters used are summarized in the ESI.† Here, we briefly discuss the relevant processes for our experiment.

As long as photon absorption can be neglected, the standing light wave acts as a pure phase grating: the interaction between the oscillating laser field and the dynamical molecular polarizability *α*_266_ imposes a periodic dipole potential onto the molecular centre-of-mass motion, which modulates the phase of the molecular matter wave along the *x*-axis:1
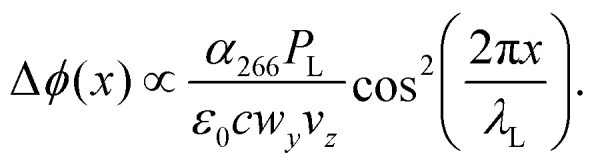
here, *P*_L_ is the laser power, *w*_*y*_ the vertical waist of the Gaussian laser beam, *v*_*z*_ the forward molecular velocity, and *c* the speed of light. Modulation of the matter wave phase results in a discrete momentum transfer to the molecule in even multiples of the photon momentum Δ*p* = ±2*nħk*_L_, where *n* ∈ ℕ and the photon wave number is *k*_L_ = 2π/*λ*_L_. This phase modulation translates into a discrete spatial distribution of the molecular density on the detector downstream. This interaction is always present, since every molecule has a finite and sometimes even a large dynamical polarisability.

The description is more involved when the molecule can also absorb at least one photon from the laser grating. In this case, it receives an additional recoil of ±*ħk*_L_ per photon. This gives rise to additional peaks exactly half way in between the diffraction orders associated with the phase grating. Even though the absorption process is probabilistic and follows a Poisson distribution, it is coherent in the sense that one cannot, not even in principle, distinguish if the photon was absorbed while it was on the way towards the mirror or back. This is due to the long coherence length (here 50 m) of our DUV laser light.^54,55^ At high intensities, absorption of *N* photons can thus disperse the molecular momentum in integer multiples of the photon momentum, Δ*p* = *nħk*_L_ with *n* = −*N*,…,*N*, and all branches of the molecular distribution associated with an even number of photons overlap at the detector position-synchronously with those affected by the phase grating alone, even though their internal state is different.

If an excited molecule decays nonradiatively, for instance, by internal conversion (IC) or intersystem crossing (ICS) to a triplet state, the momentum transfer to the molecule is determined by the phase and absorption component alone. However, if spontaneous fluorescence is emitted near the grating, this adds another momentum kick. Since the direction of spontaneously emitted photons is isotropically distributed, fluorescence would show up as a broadening of the diffraction peaks. Multiple absorption-relaxation cycles are conceivable, given the range of absorption cross sections and the laser intensities in our experiment.

Finally, the energy of a single or several photons may suffice to cleave the molecule. Our design and synthesis of ZnPc–NBE_4_ was based on the idea that molecules should be selectively removed from the molecular beam upon photo-cleavage in the antinodes of the light grating and the fragments would be kicked to beyond the acceptance angle of the fluorescence detector (0.5 mrad).

## 3 Results and discussion

**Fig. 3:**
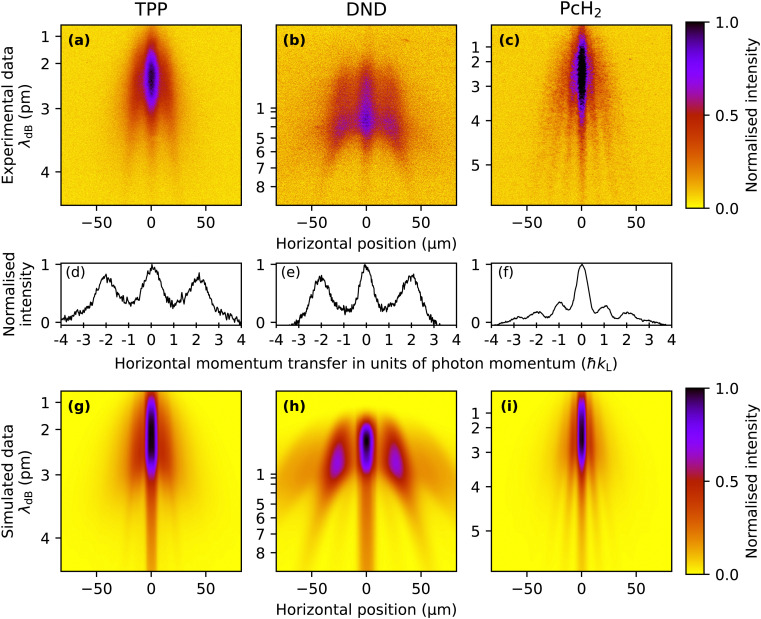
Top row: Fluorescence micrographs of the molecular interferograms: While the phase grating character dominates for TPP (a), photon absorption gains importance for phthalocyanine (c). Middle row: Normalised traces for each of the fluorescence micrographs above, rescaled to the same momentum transfer and integrated over de Broglie wavelengths larger than 3.5 pm, for which the diffraction peaks are well separated. Bottom row: The numerical simulation shows good agreement with the experiment and allows corroborating the molecular ultraviolet polarizability and absorption cross section. The vertical extents of the experimental images shown in the top row correspond to a size of 330 μm on our detection screen. The simulated images are vertically aligned and rescaled to match the de Broglie wavelengths of the corresponding experimental images. The de Broglie wavelengths given on the vertical axis are extracted by fitting the expected gravity-induced velocity distribution to the observed fringe spacing. Note that the velocities of DND in the upper half of (b) are too high to allow effective velocity selection, so the de Broglie wavelength scale is tentative in this region, and the diffraction peaks appear vertical.

The TPP diffraction pattern, as shown in Fig. 3(a), encompasses molecular velocities from approximately 150 to beyond 450 ms^−1^ which are dispersed on the detector due to their free fall in the gravitational field. Based on the extracted de Broglie wavelength *λ*_dB_ = *h*/(*mv*) ≈ 2 pm to 4 pm and the grating period *d* = *λ*_L_/2, we can attribute the observed diffraction to the effect of a pure dipole phase grating (Δ*p* = *n*2*ħk*_L_). This is in agreement with our simulation of this molecule shown in Fig. 3(g), which gives a good reproduction of the experimental results with a relatively low absorption cross section of *σ*_266_ ≈ 3 × 10^−17^ cm^2^ and a polarisability of |*α*_266_| ≈ 24 Å^3^ 4π*ε*_0_, which makes the phase grating effects dominant for these molecules. To search for an effect of photo absorption and emission, we studied DND, as shown in Fig. 3(b). The wider separation of the fringes is due to the smaller molecular weight and the consequently larger de Broglie wavelength. Also here we only observe clear diffraction peaks at positions corresponding to even multiples of *ħk*_L_, suggesting that the phase grating effect dominates for this molecule as well. This was corroborated by our simulations with parameters *σ*_266_ ≈ 1 × 10^−17^ cm^2^ and |*α*_266_| ≈ 35 Å^3^ 4π*ε*_0_ reproducing the shape of this diffraction pattern as shown in Fig. 3(h). Because the contribution of absorption is negligible for this molecule, fluorescence near the grating does not play a role here either.

In contrast to this, the result for phthalocyanine shown in Fig. 3(c) demonstrates that single-photon recoil appears as peaks of the transverse momentum at ±*nħk*_L_. Optimizing our simulations for Phthalocyanine (Fig. 3(i)) to match our experimental data suggests an absorption cross section of the order of *σ*_266_ ∼ 1 × 10^−16^ cm^2^. In contrast to this, the dipole polarizability seems to be an order of magnitude smaller than for the molecules discussed previously. This explains the larger influence of absorption in this case. The width of all diffraction fringes is comparable, indicating that fluorescence in the grating plays a minor role for PcH_2_.

**Fig. 4:**
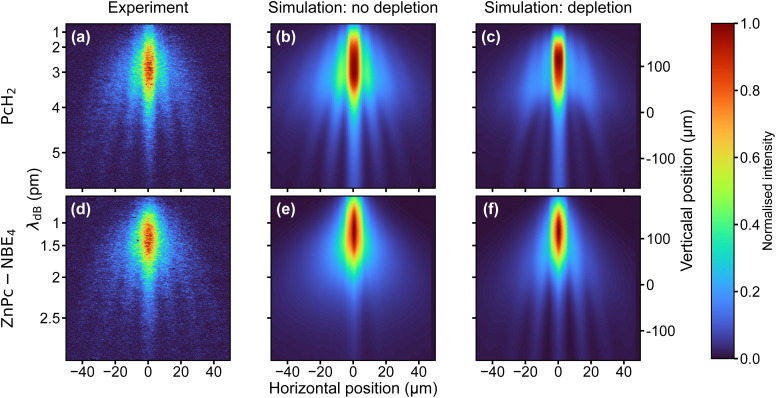
Comparing the experimental and simulated diffraction patterns of PcH_2_ (top row) and ZnPc–NBE_4_ (bottom row). The measured patterns (a) and (d) are nearly identical, which would also be expected if cleavage of ZnPc–NBE_4_ occurred in the grating. This is demonstrated by the simulation considering the effects of depletion for ZnPc–NBE_4_ ((e) and (f)), where only the latter (assuming efficient depletion) reproduces the measured data. Note that the opposite is true for PcH_2_.

Because of their absorption properties, our phthalocyanine derivatives decorated with four photoreactive *ortho*-nitroso benzaldehyde (NBE) groups are interesting for photocleavage studies. Earlier studies have shown that a photoreaction can selectively release an NBE group from a protein in the gas phase.^56^ Interestingly, we find that the molecular diffraction pattern for ZnPc–NBE_4_ is almost identical to that of PcH_2_, as shown in Fig. 4. This invites two complementary interpretations:

First, numerical simulations with and without photodissociation of ZnPc–NBE_4_ show that the fringe pattern can be explained under the assumption that photocleavage is present and efficient (Fig. 4(f)). This is true regardless of how many functional groups split off, as long as only intact parent molecules make it to the detector and all fragments are kicked beyond the detector acceptance angle. The diffraction pattern is expected to look similar to that of PcH_2_ because effective cleavage through single-photon absorption would remove the absorption peaks in the diffraction pattern. Thus, the *nħk* peaks of PcH_2_ without photocleavage (Fig. 4(a and b)) would be practically co-located with the 2*nħk* peaks of ZnPc–NBE_4_, with nearly double mass. However, a second interpretation is also attractive: at a temperature of 400 °C all four NBE groups and the coordinated Zn atom may already be detached inside the thermal source. In this case, the diffraction patterns look identical because the molecules are nearly identical.

To distinguish between these two possibilities, one can envisage two tests, one based on matter-wave arguments and one using mass spectrometry. Even though the peaks are co-located, the intensity distribution of the interference fringes should depend on the optical polarizability of the arriving molecules - which is substantially bigger for ZnPc–NBE_4_ than for PcH_2_. However, since DUV polarizabilities in the gas phase are not available from independent measurements, this interesting route remains closed for now. Collecting the emitted molecules on a glass slide behind the oven and post-analyzing them in MALDI-MS shows that thermal decomposition is almost complete - including all NBE subgroups up to the bare phthalocyanine core, as discussed in Section S3 of the ESI.† Thermal fragmentation thus precedes the optical dissociation, underlining the high sensitivity of the NBE groups to the addition of internal energy. Since similar molecules are known to survive ultrafast laser evaporation when injected into a cooling carrier gas or during electrospray ionization, photocleavage is still a promising basis for a deep ultraviolet beam splitter. The same effects and the theory will apply as described above. This insight opens a path for future explorations of peptide and protein interferometry.

## 4 Conclusion

We have shown that a standing deep-ultraviolet light wave can act as a versatile beam splitter for organic molecules. This opens the door to the manipulation of novel particles and allows acquiring new information on photophysical processes in molecules in the gas phase. Compared to typical spectroscopy methods, the deactivation process is not encoded in the final-state population, but in the molecular center-of-mass motion, *i.e.*, the spatial diffraction pattern, where we can detect each molecule in principle with a single-molecule sensitivity.^50^ The availability of a rich set of internal states will allow us to explore a variety of photophysical and photochemical effects for future beam splitters and molecular analysis: For instance, when molecules are optically excited to long-lived triplet states, beam deflection in a magnetic field can be sensitively read out from interference patterns. Similarly, photoisomerization in the DUV grating will serve as a measurement-induced beam splitter when the detector is sensitive to molecular conformers. We envisage that intense deep UV light gratings will become important building blocks for many all-optical matter-wave interferometers, designed to explore molecular quantum optics in the regime of high mass and high complexity.

## Author contributions

Conceptualization: MA, CB, KS, RF, MM. Formal analysis: LM, BS, KH, RF. Materials and synthesis: ADS, MM. Funding acquisition: MA, KH, MM. Investigation: KS, RF, LM, CB. Methodology: all authors. Software: LM, RF. Supervision: MA, CB, KH, BS, MM. Writing – original draft: MA, CB, KS, RF.

## Data availability

Data for this article and ESI,† including raw, background-corrected and preprocessed diffraction images, as well as data files for simulated images, are available in the zenodo repository under https://doi.org/10.5281/zenodo.13124328.

## Conflicts of interest

There are no conflicts to declare.

## Supplementary Material

CP-026-D4CP03059A-s001

CP-026-D4CP03059A-s002

CP-026-D4CP03059A-s003

CP-026-D4CP03059A-s004

CP-026-D4CP03059A-s005
