# Supplementary material for: Diffracting molecular matter-waves at deep-ultraviolet standing-light waves
Source: Phys Chem Chem Phys. 2024 Oct 22;26(43):27617–23. doi: 10.1039/d4cp03059a (PMC11514965; doi:10.1039/d4cp03059a)
Supplement: CP-026-D4CP03059A-s001 [file CP-026-D4CP03059A-s001.pdf]

Supplementary Information (SI) for Physical Chemistry Chemical Physics.
This journal is © the Owner Societies 2024

PCCP

ARTICLE TYPE

**Electronic Supplementary Information (ESI)**

# Diffracting molecular matter-waves at deep-ultraviolet standing-light waves

Ksenija Simonović,[a*] Richard Ferstl,[a] Alfredo Di Silvestro,[b] Marcel Mayor, [b] Lukas Martinetz,[c] Klaus Hornberger,[c] Benjamin A. Stickler,[d] Christian Brand,[e] and Markus Arndt[a‡]

## 1 Simulations

The complete quantum theory for this experiment is provided in a separate manuscript[1]. It includes all the experimental parameters shown in Fig. 1 and listed in Table 1.

When we integrate the fluorescence signal along the horizontal axis and plot it as a function of the vertical axis, it should represent the velocity distribution, as fast molecules arrive at the top and slow molecules at the bottom of the screen. From a fit to the experimental data, we determine the position of the velocity selection slit $y_{02}$ as well as the offset $p_{0,z}/m$

The vertical position of the light grating $y_{0,g}$, the molecular UV polarizability $\alpha_r$ and the absorption cross section $\sigma$ are found from a least-square fit of the simulation to the experiment, performed over all pixels of the screen. The following parameters enter the simulation: We assume fluorescence to be negligible $\phi_F = 0$, a perfect mirror reflectivity ($\eta = 1$) and that there is no photodepletion. Internal conversion can be neglected compared to intersystem crossing $\phi_{IC} = 0$, $\phi_{ISC} = 1$. The gravitational acceleration is $g = -9.81\ \mathrm{m/s^2}$ and the local rotation frequency of the Earth is $\boldsymbol{\omega}\cdot\mathbf{e}_x = 5.4\times10^{-5}\mathrm{s}^{-1}$, and $\boldsymbol{\omega}\cdot\mathbf{e}_y = -4.9\times10^{-5}\mathrm{s}^{-1}$. The detection screen is discretized in squares of width $d_{px} = 0.33\,\mu$m, which is the calibrated size of the pixels recorded by the camera. The images are normalized to unity. In the case of DND, the optimization procedure is impeded by a vertical bias of the signal, most likely caused by inhomogeneous illumination of the diffraction image. We set the theory parameters to reproduce the features of the observed diffraction image.

The simulation allows reproducing the experimental data and identifying the dominant diffraction processes. In Fig. 2 we show the range of molecular polarizabilities and absorption cross sections that fit the experiment. The heat map shows the natural logarithm of the residual sum of squares of differences between the simulation and the experimental data. This permits us to estimate the deep ultraviolet polarizability and absorption cross sections. Note that the diffraction patterns are insensitive to the sign of polarizability in this set-up.

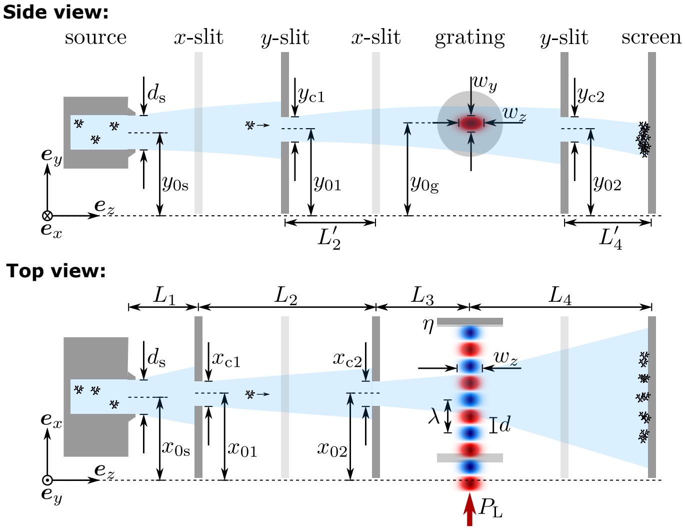


Fig. 1 Schematic depiction of the experimental setup with all simulation-relevant parameters as listed in Tab. 1 and the ESI text.

## 2 Synthesis and characterisation of ZnPc−NBE$_4$

**General Procedures:** All commercially available chemicals were used without further purification. Dry solvents were used as crown cap and purchased from ACROS ORGANICS and SIGMA-ALDRICH. NMR solvents were obtained from CAMBRIDGE ISOTOPE LABORATORIES, INC. (Andover, MA, USA) or SIGMA-ALDRICH. All NMR experiments were performed on the BRUKER AVANCE III or III HD, two- or four-channel NMR spectrometers operating at 400.13, 500.13 or 600.27 MHz proton frequency. The instruments were equipped with direct observer BBFO, indirect BBI, or cryogenic four-channel QCI (H/C/N/F) 5 mm probes, all with self-shielded z-gradient. The experiments were performed at 298 K or 295 K. All chemical shifts ($\delta$) are reported in parts per million (ppm) relative to the solvent used, and coupling constants

[a] University of Vienna, Faculty of Physics, VDS, VCQ, Boltzmanngasse 5, 1090 Vienna, Austria
[b] Department of Chemistry, University of Basel, St. Johannsring 19, 4056 Basel, Switzerland
[c] University of Duisburg-Essen, Lotharstraße. 1, 47048 Duisburg, Germany
[d] University of Ulm, Institute for Complex Quantum Systems, Albert-Einstein-Allee 11, 89069 Ulm, Germany
[e] German Aerospace Center (DLR), Institute of Quantum Technologies, Wilhelm-Runge-Straße 10, 89081 Ulm, Germany
* ksenija.simonovic@univie.ac.at
‡ markus.arndt@univie.ac.at

| Parameter | TPP | $PcH_2$ | DND |
|---|---|---|---|
| Laser power $P_L$ | 0.92 W | 0.96 W | 1.0 W |
| Laser waist $w_y$ | 13 $\mu$m | 16 $\mu$m | 20 $\mu$m |
| Molecule mass $m$ | 614.74 u | 514.5 u | 290.3 u |
| DUV polarisability $\lvert\alpha_{266}\rvert$ | $24\,\text{Å}^3 \cdot 4\pi\varepsilon_0$ | $1.2\,\text{Å}^3 \cdot 4\pi\varepsilon_0$ | $35\,\text{Å}^3 \cdot 4\pi\varepsilon_0$ |
| DUV absorption cross section $\sigma_{266}$ | $3.4 \times 10^{-21}$ m$^2$ | $8.5 \times 10^{-21}$ m$^2$ | $1 \times 10^{-21}$ m$^2$ |
| Grating height $y_{0g}$ | −12.7 $\mu$m | −5.1 $\mu$m | −43.0 $\mu$m |
| Slit height $y_{02}$ | −21.6 $\mu$m | −16.5 $\mu$m | −54.7 $\mu$m |
| Source temperature $T$ | 688 K | 746 K | 539 K |
| Slit width $x_{c1}$ | 3.5 $\mu$m | 2.7 $\mu$m | 5.0 $\mu$m |
| Slit width $x_{c2}$ | 1.7 $\mu$m | 0.6 $\mu$m | 3.0 $\mu$m |
| Momentum shift $p_{0,z}/m$ | 117 m s$^{-1}$ | 76.5 m s$^{-1}$ | 10.2 m s$^{-1}$ |
| Source size $d_s$ | 200 $\mu$m | | |
| Slit position $y_{01}$ | 0 $\mu$m | | |
| Slit width $y_{c1}$ | 1 m * | | |
| Slit width $y_{c2}$ | 20 $\mu$m | | |
| Distance $L_1$ | 0.52 m | | |
| Distance $L_2$ | 0.3 m | | |
| Distance $L_2'$ | 0.02 m | | |
| Distance $L_3$ | 0.08 m | | |
| Distance $L_4$ | 0.69 m | | |
| Distance $L_4'$ | 0.605 m | | |
| Grating period $d$ | 133 nm | | |

Table 1 Parameters used to simulate the diffraction images shown in Fig. 3 of the main text. * Slit $y_{c1}$ is not present in our experimental configuration.

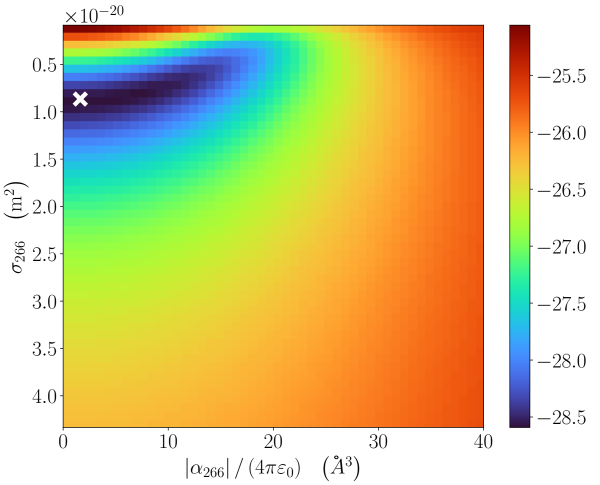


Fig. 2 Natural logarithm of the residual sum of squares as a function of the parameters $\lvert\alpha_{266}\rvert$ and $\sigma_{266}$ computed for the simulated and experimental data of $PcH_2$. Lower values indicate better agreement between the two datasets. The minimum is marked by a white cross.

(J) are given in Hertz (Hz). The multiplicities are written as: s = singlet, d = doublet, t = triplet, dd = doublet of doublet, m = multiplet. Flash column chromatography (FCC) was performed with SiliaFlash® P60 from SILICYCLE with a particle size of 40 $\mu$m to 63 $\mu$m (230-400 mesh), and for the TLC silica gel 60 F254 glass plates with a thickness of 0.25 mm from Merck were used. The detection was carried out using a UV lamp at 254 or 366 nm. UV/VIS absorption spectra were recorded on a Jasco V-770 Spectrophotometer with a 1 cm quartz glass cuvette. High-resolution mass spectra (HRMS) were measured with a Bruker Maxis 4G ESI-TOF instrument or a Bruker solariX spectrometer with a MALDI source.

**Synthetic steps to the target structure:** As displayed in Scheme 1:, the target structure ZnPc−$NBE_4$ was assembled in two synthetic steps, followed by purification via sublimation. As the first step, the condensation between (2-nitrophenyl)methanol and 4-hydroxyphthalonitrile provided the phthalonitrile precursor (4-((2-nitrobenzyl)oxy)phthalonitrile) exposing the photo-cleavable nitro benzyl ether subunit. Its subsequent cyclotetramerization in the presence of zinc acetate provided the target structure as mixture of regioisomers. Sublimation of the crude mixture provided the highly symmetric regioisomer **ZnPc−$NBE_4$** which was used in the experiments reported here.

**Synthesis of 4-((2-nitrobenzyl)oxy)phthalonitrile:** An oven dried 100 mL flask under argon was charged with (2-nitrophenyl)methanol (1831 mg, 11.6 mmol, 1.0 eq.), 4-hydroxyphthalonitrile (2006 mg, 13.9 mmol, 1.2 eq.), triphenylphosphine (4564 mg, 17.4 mmol, 1.5 eq.) and dry tetrahydrofuran (THF, 50 mL). The reaction mixture was cooled to 0 °C and diisopropyl azodicarboxylate (DIAD, 3.43 mL, 17.4 mmol, 1.5 eq.) was added drop wise. The reaction was warmed up to room temperature and stirred for 12 hours. After TLC confirmed full conversion of the starting materials the solvent was removed under reduced pressure and the remaining residue was purified by column chromatography (ethyl acetate/cyclohexane = 1/2) to yield the desired product as an off-white solid (2000 mg, 11.6 mmol, 61 %).

**Analytical data for 4-((2-nitrobenzyl)oxy) phthalonitrile:** $^1$H NMR (400 MHz, DMSO−$d_6$, 22 °C) $\delta$ = 8.18 (dd, J = 8.2, 1.3 Hz, 1H), 8.08 (d, J = 8.8 Hz, 1H), 7.91 (d, J = 2.7 Hz, 1H), 7.81 (td, J = 7.6, 1.3 Hz, 1H), 7.74 (dd, J = 7.8, 1.5 Hz, 1H), 7.66

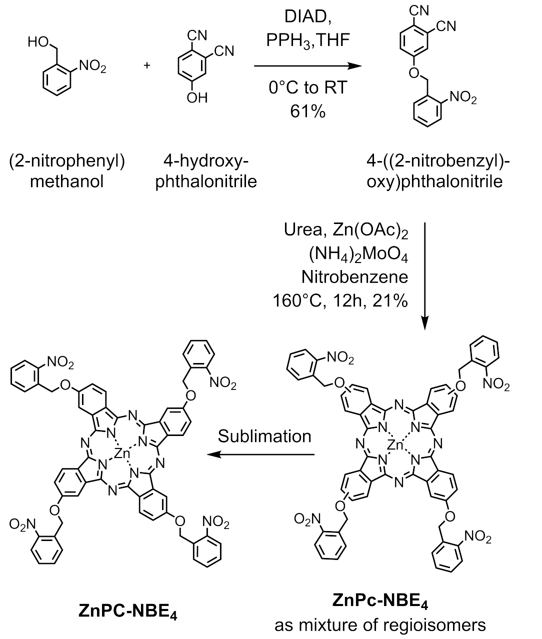


Scheme 1 Synthesis scheme ZnPc−$NBE_4$.

(ddd, J = 8.7, 7.4, 1.5 Hz, 1H), 7.57 (dd, J = 8.9, 2.6 Hz, 1H), 5.66 (s, 2H). $^{13}$C NMR (101 MHz, DMSO-$d_6$, 22°C) $\delta$ = 161.18, 147.41, 135.92, 134.25, 131.07, 129.57, 129.21, 125.08, 120.60, 120.44, 116.37, 116.10, 115.63, 106.69, 67.51 ppm. HRMS (ESI-ToF, MeOH, positive mode): calc. for $[C_{60}H_{36}N_{12}O_{12}Zn]^+$ 302.0536; found 302.0537.

**Synthesis of ZnPc−$NBE_4$:** An oven dried 50 mL round-bottomed flask under argon was charged with 4-((2-nitrobenzyl)oxy)phthalonitrile (2000 mg, 7.16 mmol, 1.0 eq.), urea (1247 mg, 20.8 mmol, 2.9 eq.), zinc acetate (328 mg, 1.79 mmol, 0.28 eq.), ammonium molybdate (28 mg, 0.14 mmol, 0.02 eq.) and were heated in nitrobenzene (30 mL) at 160 °C for 12 h. After cooling, the reaction mixture was treated with water and the green dark product that precipitated was filtered off, successively washed with water, ethyl acetate and ethanol. After evaporation of the solvent under reduced pressure, the crude was purified by column chromatography (acetone/pyridine 5/1 and later 1/1) to obtain the mixture of regioisomers as a blue solid (460 mg, 1.79 mL, 21 %). Sublimation provided the pure regioisomer in yields below 5 %.

**Analytical data for ZnPc−$NBE_4$**: $^1$H NMR (500 MHz, DMSO−$d_6$, 22 °C) $\delta$ = 9.25 (d, J = 6.8 Hz, 4H), 8.91 (d, J = 20 Hz, 4H), 8.29 (t, J = 7.4 Hz, 4H), 8.19 (d, J = 7.5 Hz, 4H), 7.79 (t, J = 7.6 Hz, 4H), 7.88 (s, 4H), 7.77 (t, J = 7.8 Hz, 4H), 6.05 (s, 8H). HRMS (MALDI, DCM/DCTB Mix 1:10, positive mode): calc. for $[C_{60}H_{36}N_{12}O_{12}Zn]^+$ 1180.1862; found 1180.1858.

## 3 MALDI mass spectrum of evaporated ZnPc-$NBE_4$

As discussed in the main text, there is evidence that the functionalized phthalocyanine may already decomposed in the source. This hypothesis is corroborated by the the MALDI spectrum taken from the evaporated and recollected molecular sample as shown in Figure 3.

## Notes and references

1 L. Martinetz, B. A. Stickler, K. Simonović, R. Ferstl, C. Brand, M. Arndt and K. Hornberger, *Probing molecular photophysics in a matter-wave interferometer*, 2024, `https://arxiv.org/abs/2407.18775v1`.

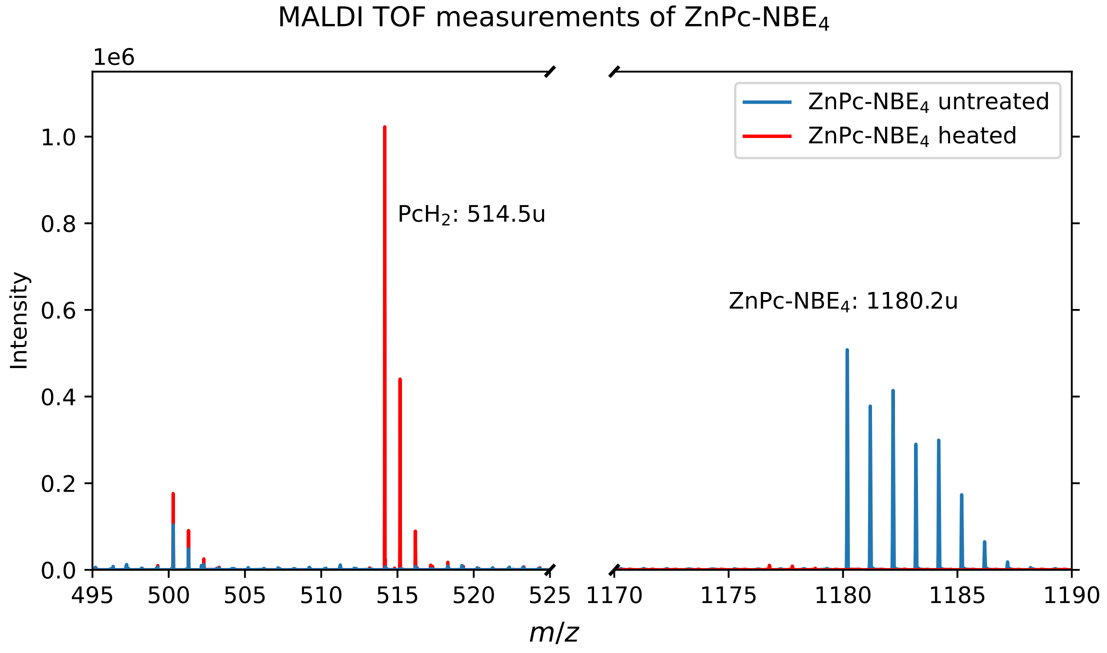


Fig. 3 MALDI-TOF measurements of untreated ZnPc−$NBE_4$ (blue) and after oven sublimation at temperatures needed to create a molecular beam. While the untreated sample shows a strong peak around 1180 u as expected, the heated one only shows a peak at 514.5 u. This is the mass of the metal-free phthalocyanine core, indicating that the sample undergoes nearly complete thermal decomposition in the source already.
