# Supplementary figures and images for: Diffracting molecular matter-waves at deep-ultraviolet standing-light waves
Source: Phys Chem Chem Phys. 2024 Oct 22;26(43):27617–23. doi: 10.1039/d4cp03059a (PMC11514965; doi:10.1039/d4cp03059a)

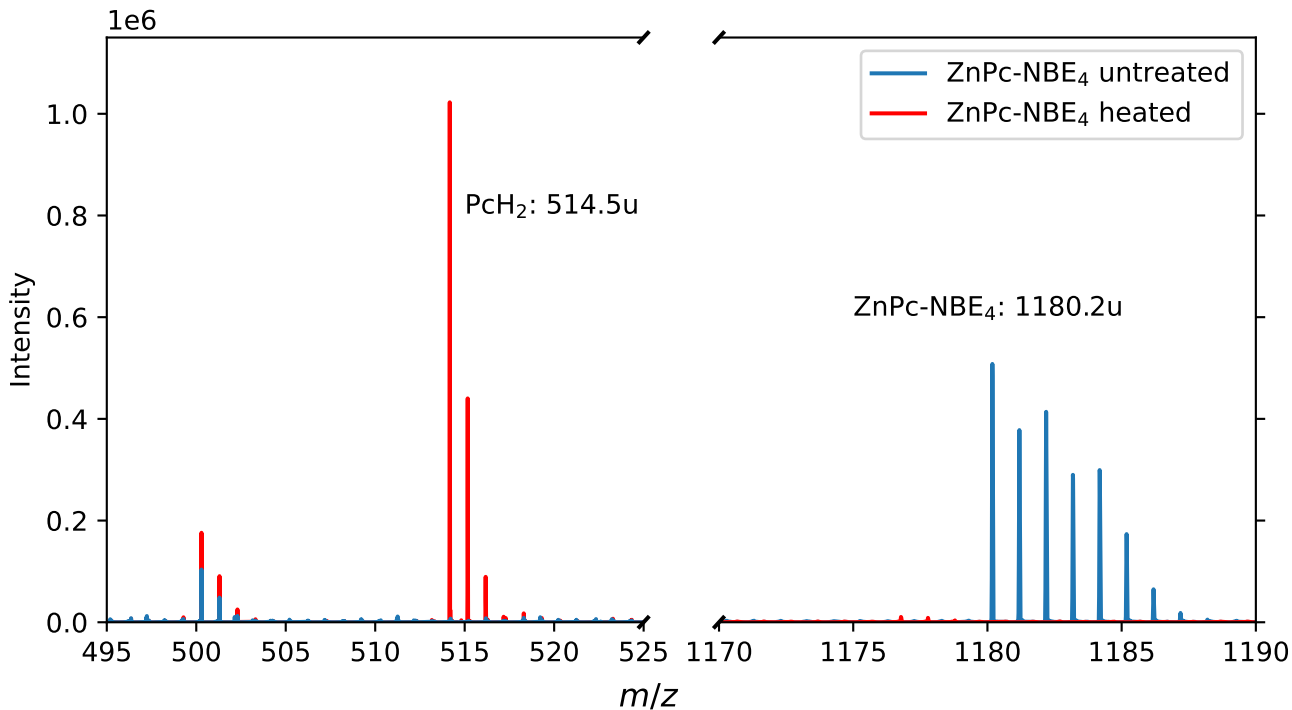

Supplement: CP-026-D4CP03059A-s002 [file CP-026-D4CP03059A-s002.pdf]

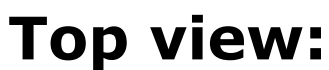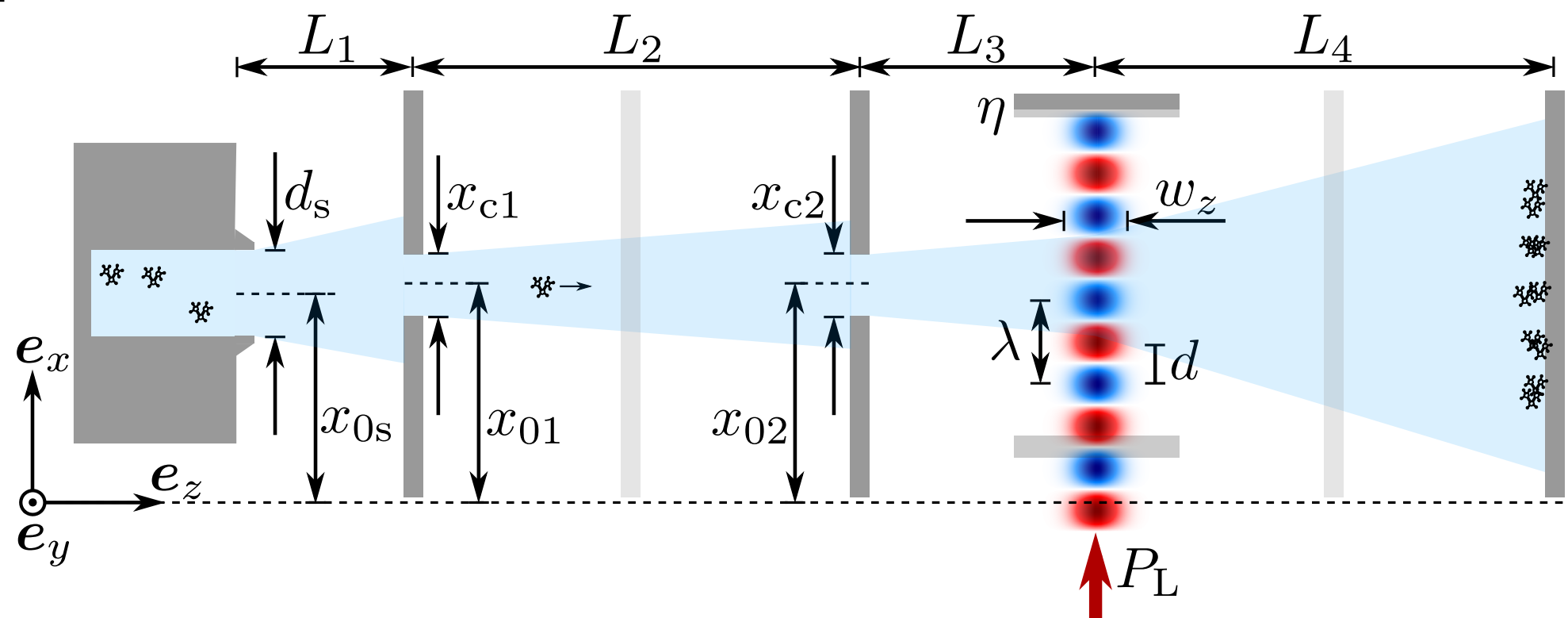

Supplement: CP-026-D4CP03059A-s003 [file CP-026-D4CP03059A-s003.pdf]

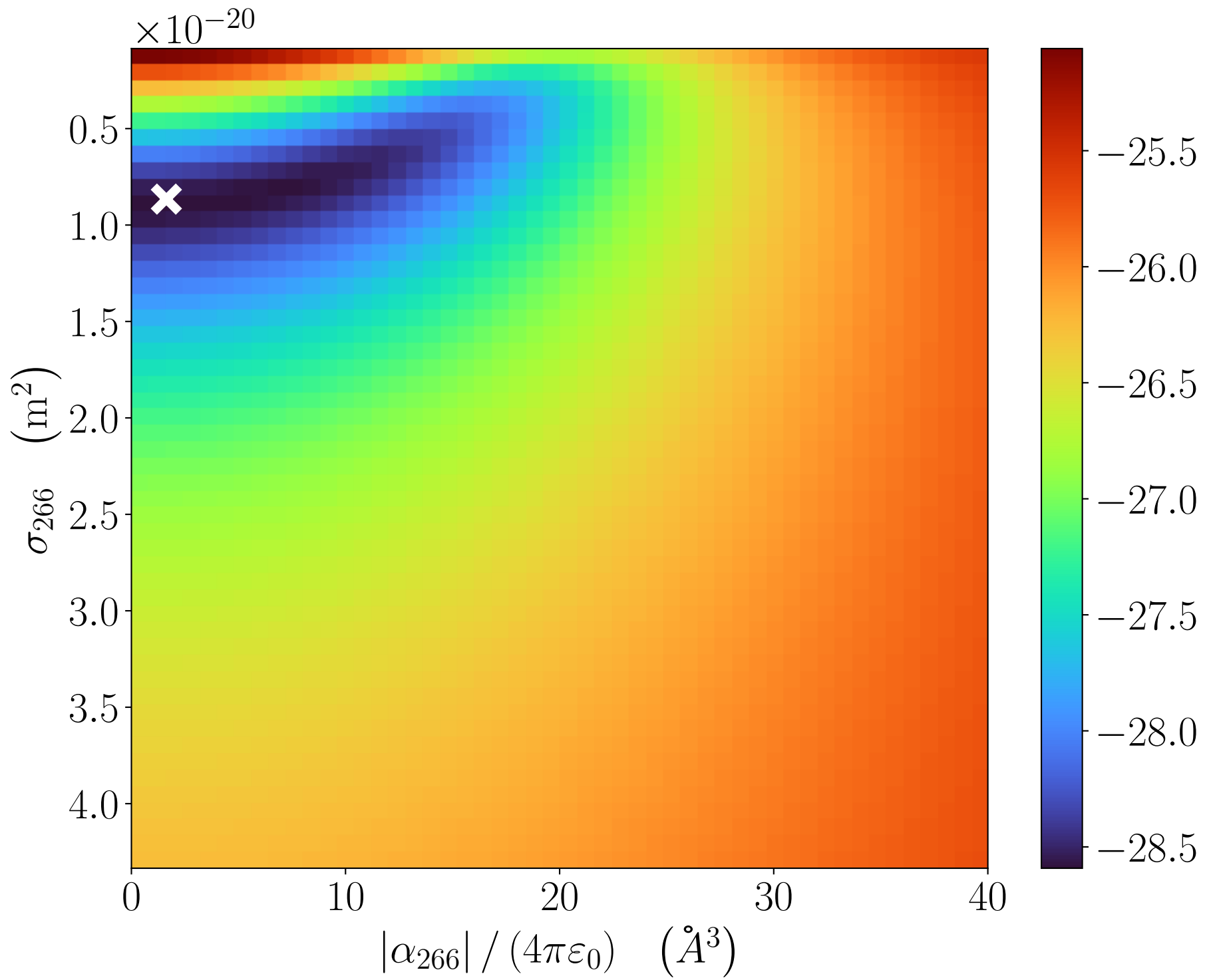

Supplement: CP-026-D4CP03059A-s004 [file CP-026-D4CP03059A-s004.pdf]

# MALDI TOF measurements of ZnPc-NBE<sub>4</sub>

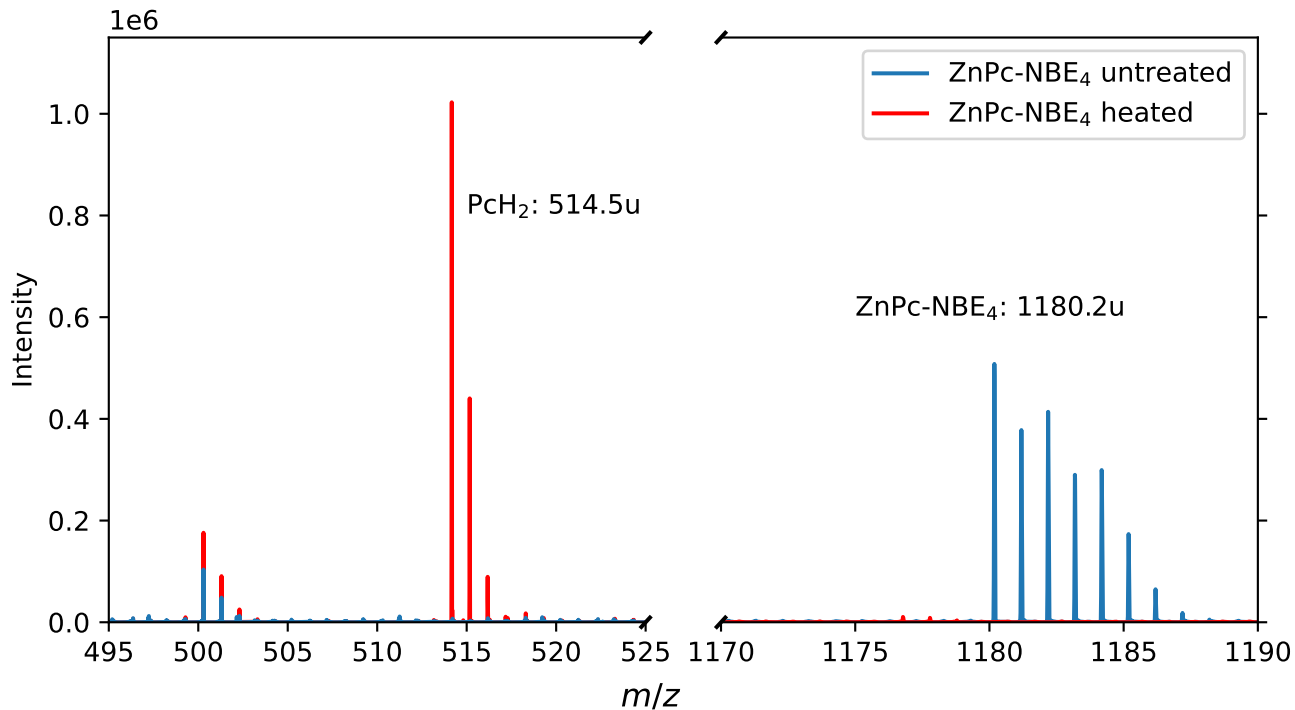

Supplement: CP-026-D4CP03059A-s005 [file CP-026-D4CP03059A-s005.zip › SI_Fig1.pdf]

**Side view:**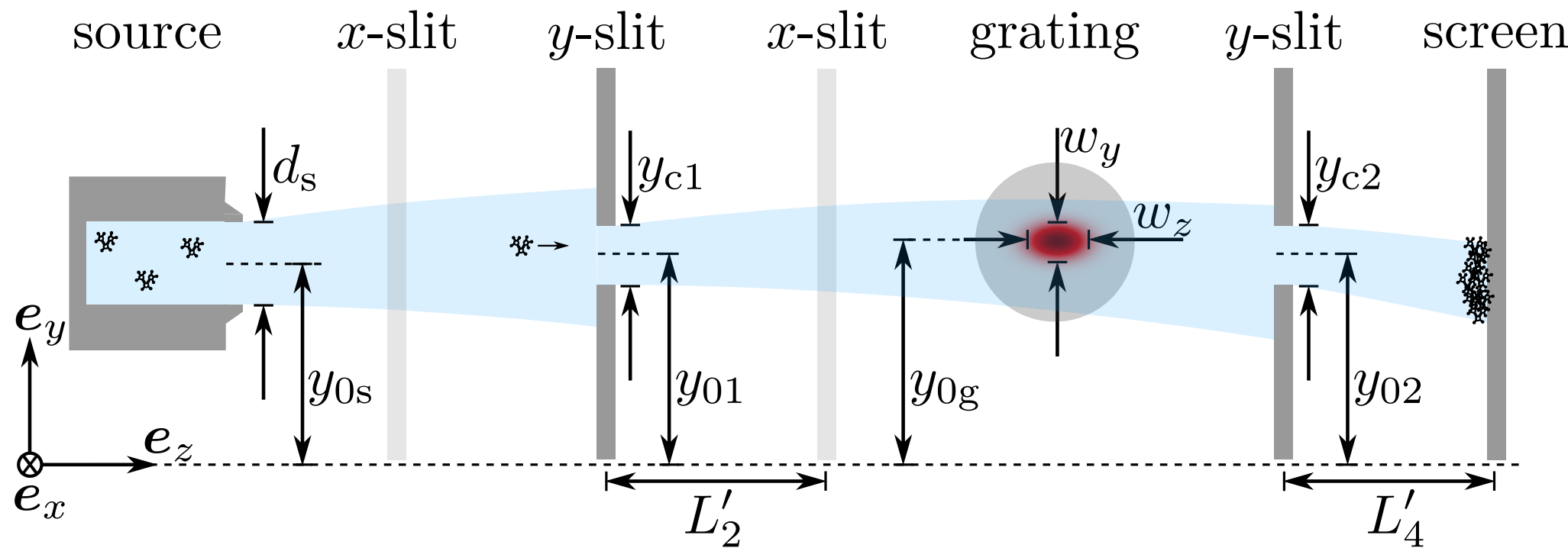**Top view:**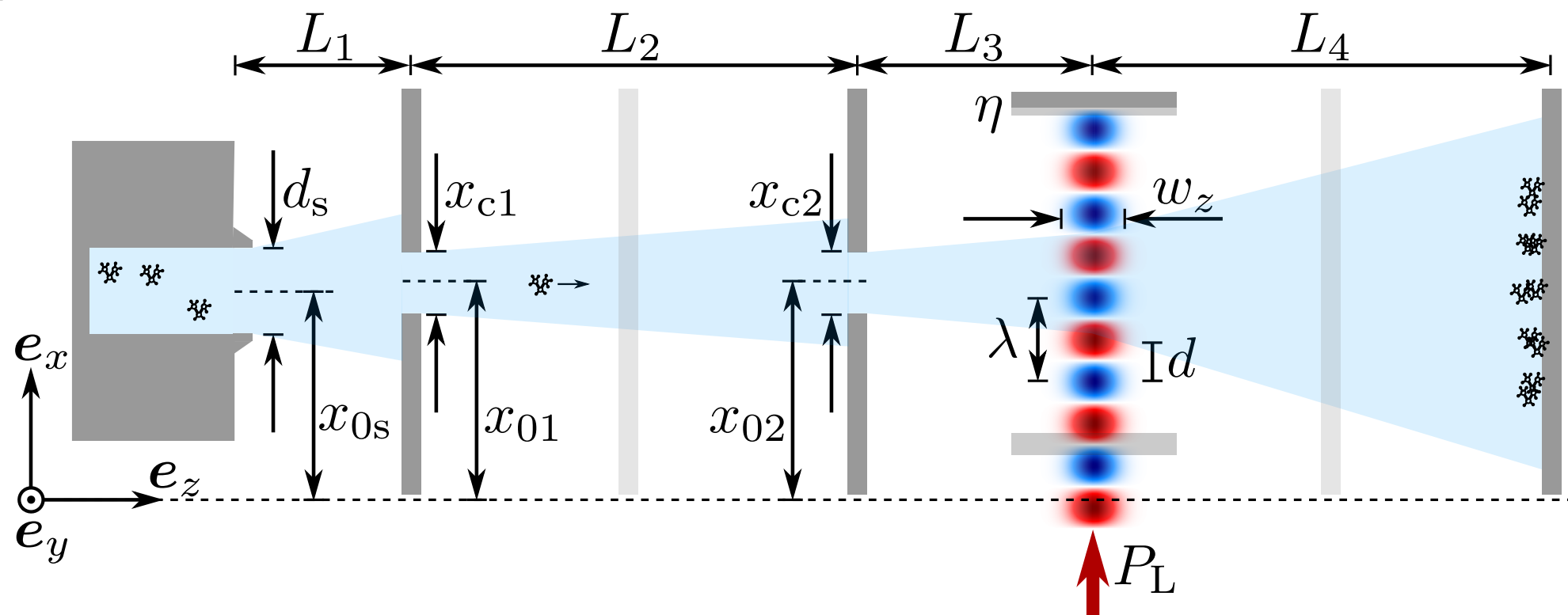

Supplement: CP-026-D4CP03059A-s005 [file CP-026-D4CP03059A-s005.zip › SI_Fig2.pdf]

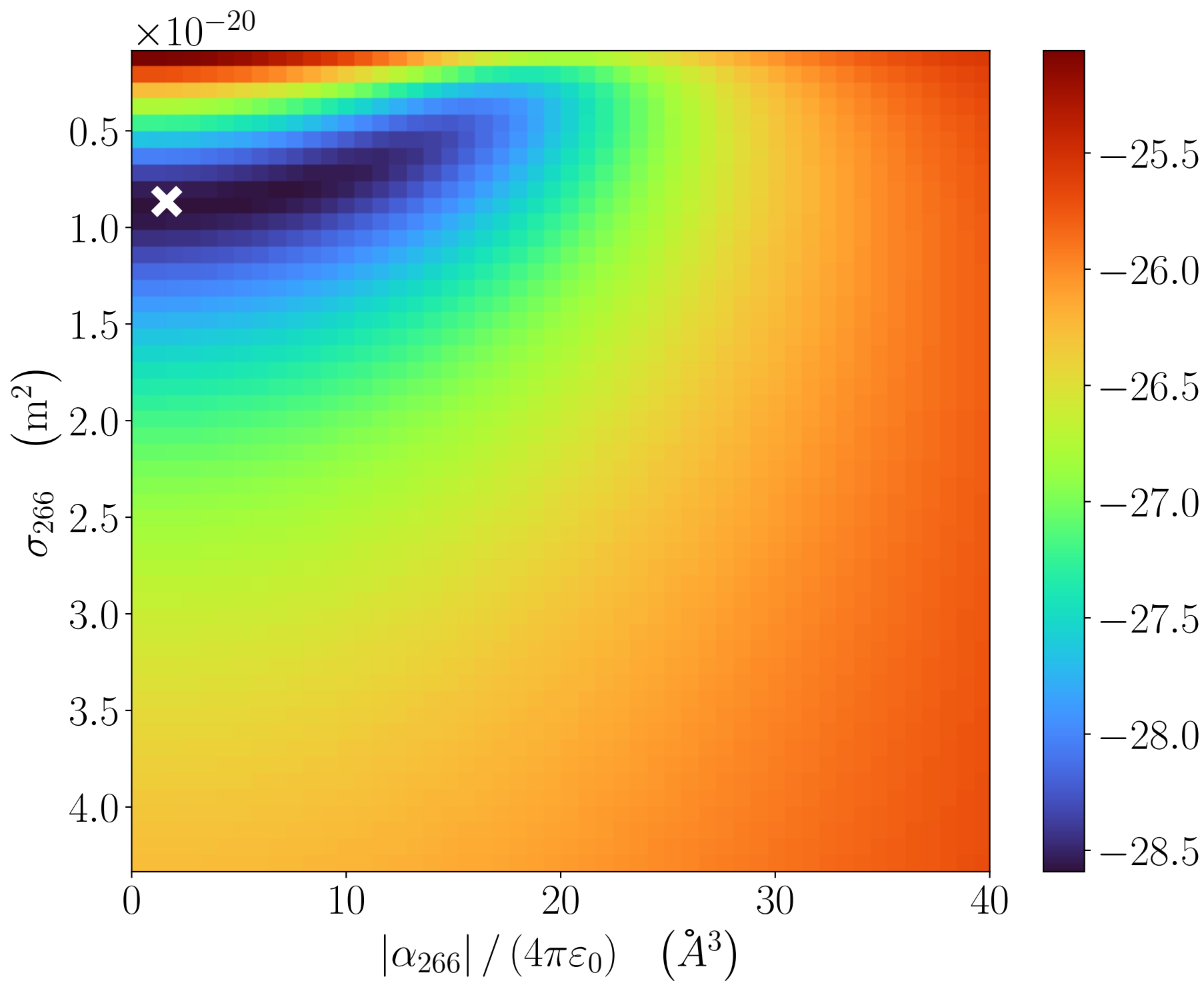

Supplement: CP-026-D4CP03059A-s005 [file CP-026-D4CP03059A-s005.zip › SI_Fig3.pdf]
